# β-Caryophyllene Ameliorates 2,4-Dinitrochlorobenzene-Induced Atopic Dermatitis through the Downregulation of Mitogen-Activated Protein Kinase/EGR1/TSLP Signaling Axis

**DOI:** 10.3390/ijms232314861

**Published:** 2022-11-28

**Authors:** Sung Shin Ahn, Hyunjin Yeo, Euitaek Jung, Sukjin Ou, Young Han Lee, Yoongho Lim, Soon Young Shin

**Affiliations:** 1 Department of Biological Sciences, Sanghuh College of Lifesciences, Konkuk University, Seoul 05029, Republic of Korea; 2 Division of Bioscience and Biotechnology, Konkuk University, Seoul 05029, Republic of Korea

**Keywords:** atopic dermatitis, β-caryophyllene, early growth response 1, keratinocyte, mitogen-activated protein kinase signaling pathway, thymic stromal lymphopoietin

## Abstract

Atopic dermatitis (AD) is one of the most common inflammatory skin diseases accompanied by severe itching. β-caryophyllene (BCP), which displays anti-inflammatory activity, is a natural agonist of cannabinoid receptor 2. However, the therapeutic effects of BCP on atopic dermatitis (AD) remain poorly understood. The current study aimed to evaluate the topical therapeutic efficacy of BCP in an AD-like mouse model. Thymic Stromal Lymphopoietin (TSLP) is a keratinocyte-derived cytokine that drives AD pathogenesis. This study also investigated the effect of BCP on the interleukin 4 (IL-4)-induced expression of TSLP in HaCaT keratinocytes. We found that the topical application of BCP alleviated AD-like skin inflammation and inhibited the infiltration of proinflammatory cells into skin lesions. Moreover, the topical application of BCP reduced EGR1 (Early Growth Response 1) and TSLP expression in AD-like skin lesions. We also found that BCP inhibited IL-4-induced TSLP expression by downregulating mitogen-activated protein kinase (MAPK)-mediated EGR1 expression in HaCaT keratinocytes. These findings demonstrate that BCP ameliorates DNCB-induced AD-like skin lesions through the downregulation of the MAPK/EGR1/TSLP signaling axis. BCP may be applicable for developing topical therapeutic agents for chronic skin inflammatory diseases, such as AD.

## 1. Introduction

Atopic dermatitis (AD) is a chronic skin disease characterized by severe itching. Excessive inflammation in AD skin impairs skin barrier function and causes itching [1]. However, the precise etiology of AD remains poorly understood and may vary between individual genetic and environmental factors. Although the pathogenic mechanisms of AD are complex, growing evidence suggests that the early onset of cutaneous inflammation in AD is strongly associated with an imbalance in innate cutaneous immunity and aberrant immune responses [1]. At the early onset, increased CD4^+^ T helper type 2 (Th2) lymphocytes, which are defined by the production of interleukin 4 (IL-4) and IL-13, constitute a primary driver in the pathogenesis of the disease [2]. Indeed, genetically engineered transgenic mice overexpressing IL-4 in their skin trigger inflammatory AD-like skin lesions, suggesting that Th2 cytokine expression plays a crucial role in AD development [3]. In the chronic phase, disease symptoms are further exacerbated by the polarization of Th1 and Th17 lymphocytes, increased production of Th1 cytokines (such as IL-2, TNFα, and IFNγ), and Th17 cytokines (IL-1β, TNFα, IL-17, IL-21, IL-22, and IL-6), and facilitate the infiltration of various inflammatory immune cells [4]. 

Thymic Stromal Lymphopoietin (TSLP) is an IL-2 cytokine family member secreted by various non-immune cells, such as epithelial cells, smooth muscle cells, fibroblasts, and epidermal keratinocytes [5]. TSLP expression is induced by several cytokines, pathogenic microbial infections, mechanical tissue injuries, and allergens [6]. It promotes the dendritic cell-mediated polyclonal expansion of T cells [7]. Thus, TSLP is considered a master regulator of type 2 immune response at the barrier surface of the skin [8]. 

During allergic inflammation, TSLP is abundantly produced in keratinocytes and promotes the differentiation of naïve Th0 cells into Th2 lymphocytes [9]. It also activates dendritic cells to expand the Th2 cell population and stimulate the secretion of Th2 cytokines and tumor necrosis factor-alpha (TNFα) from infiltrating effector T cells [10,11]. TSLP also potentially activates mast cells and eosinophils to produce high levels of Th2-like cytokines, further promoting allergic inflammation [12]. Additionally, TSLP directly stimulates sensory neurons to trigger a Th2-independent itch sensation [13]. Furthermore, the overexpression of TSLP in keratinocytes in a genetically engineered mouse model led to AD-like skin inflammatory responses [14]. These findings suggest that TSLP triggers both the initiation and maintenance of AD by activating Th2-mediated allergic inflammatory responses, implicating TSLP as a crucial mediator in the pathogenesis of AD [15]. Therefore, targeting TSLP may provide a potential therapeutic strategy for AD treatment.

**Figure 1:**
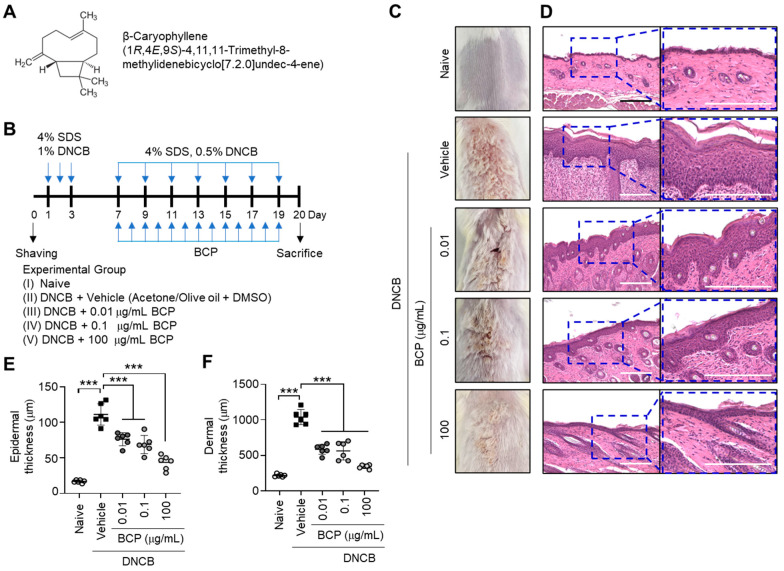
Effect of topical administration of BCP on the AD-like skin lesions in BALB/c mice. (**A**) Chemical structure of BCP. (**B**) The experimental schedule for DNCB and BCP treatments. (**C**) Representative images of the dorsal skin of BALB/c mice. (**D**) Paraffin-embedded skin tissue sections were prepared on day 20 and performed H&E staining. The enlarged region is provided in the dotted box. Scale bars, 200 μm. (**E**,**F**) The measurement of epidermal (**E**) and dermal (**F**) thickness using ImageJ. Error bar, mean ± SD (*n* = 6). *** *p* < 0.001. Naive, untreated control; DNCB, 2,4-dinitrochlorobenzene; BCP, β-caryophyllene; DMSO, dimethylsulfoxide; H&E, hematoxylin and eosin staining.

Cannabinoid (CB) receptors are G-protein-coupled 7-transmembrane proteins coupled with Gi/o subunits. CB receptors are two subtypes, type 1 (CB1) and type 2 (CB2). CB1 and CB2 receptors are predominantly found in neuronal tissues and peripheral lymphoid tissues/immune cells, respectively [16]. CB1 signaling is involved in the modulation of ion channel function related to cognition, memory, and learning, whereas CB2 receptors play a role in anti-inflammatory responses [17]. β-Caryophyllene (BCP, CAS:87-44-5, *trans*-4,11,11-trimethyl-8-methylenebicyclo[7,2,0]undec-4-ene; Figure 1A) is a naturally occurring bicyclic sesquiterpene compound that acts as a natural agonist of the CB2 receptor [18]. Previous studies have demonstrated that BCP inhibits the production of proinflammatory cytokines in immune cells [18] and exerts anti-inflammatory effects in vivo [19,20]. Based on these findings, we hypothesized that BCP, with its anti-inflammatory properties, might have beneficial effects when used as a topical therapeutic agent for AD treatment.

This study aimed to evaluate the therapeutic efficacy of the topical application of BCP in vivo in AD animal models and to define the underlying mechanisms in HaCaT keratinocytes. We found that AD-like skin lesions sensitized with 2,4-dinitrochlorobenzene (DNCB) were markedly alleviated by the topical application of BCP. IL-4 is a Th2-derived cytokine central to AD pathogenesis [3]. TSLP is a crucial mediator of the pathogenesis of AD [15]. In this study, we demonstrate that IL-4-induced TSLP expression was inhibited by BCP through downregulating early growth response 1 (EGR1) expression in HaCaT keratinocytes. 

## 2. Results

### 2.1. β-Caryophyllene (BCP) Alleviated 2,4-Dinitrochlorobenzene (DNCB)-Induced Atopic Dermatitis-like Skin Lesions in BALB/c Mice

To evaluate the therapeutic effect of BCP in experimental AD in vivo, we sensitized BALB/c mice with sodium dodecyl sulfate (SDS) and 2,4-dinitrochlorobenzene (DNCB) on the back skin, with or without BCP, for 20 days (Figure 1B). The back skin of DNCB-challenged mice showed AD-like skin lesions, such as superficial erosion (Figure 1C). Hematoxylin and eosin (H&E) staining demonstrated that BCP attenuated DNCB-induced epidermal hyperplasia (Figure 1D). Notably, the application of topical BCP substantially reduced DNCB-induced thickening of the epidermis (Figure 1E) and dermis (Figure 1F) in a dose-dependent manner. These data suggest that the topical administration of BCP relieves DNCB-induced skin inflammation.

### 2.2. Topical Application of BCP Inhibits DNCB-Induced Infiltration of Inflammatory Cells in AD-like Skin Lesions 

**Figure 2:**
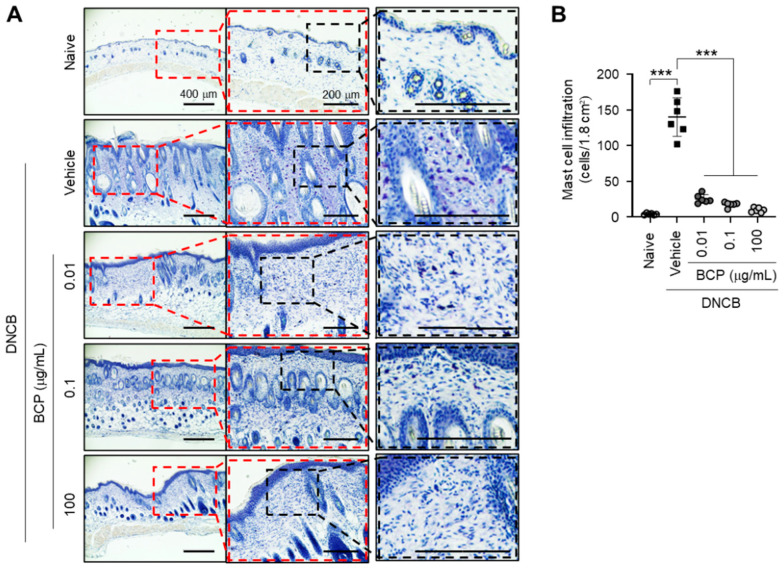
Effect of topical administration of BCP on mast cell infiltration in skin lesions. (**A**) Mast cells were stained with 0.1% toluidine blue. The blue spots indicate the infiltrated mast cells. Scale bars, 400 μm or 200 μm (enlarged images). (**B**) The number of mast cells per 1.8 cm^2^ was counted. Error bar, mean ± SD (*n* = 6). *** *p* < 0.001. Naive, untreated control; DNCB, 2,4-dinitrochlorobenzene; BCP, β-caryophyllene.

One of the pathological features of AD-like skin lesions exposed to DNCB is an increase in the infiltration of various proinflammatory cells into the dermis [21]. We determined the effect of BCP on mast cell infiltration by histological analysis using toluidine blue (TB) staining (Figure 2A). Topical application of BCP significantly (*p* < 0.001) reduced infiltrated TB-positive cells (Figure 2B). These data suggest a beneficial effect of topical BCP in ameliorating AD-like skin inflammation by reducing the infiltration of proinflammatory cells into the site of inflammation in a mouse model.

### 2.3. IL-4-Induced TSLP Expression Is Attenuated by BCP at the mRNA Level in HaCaT Keratinocytes

**Figure 3:**
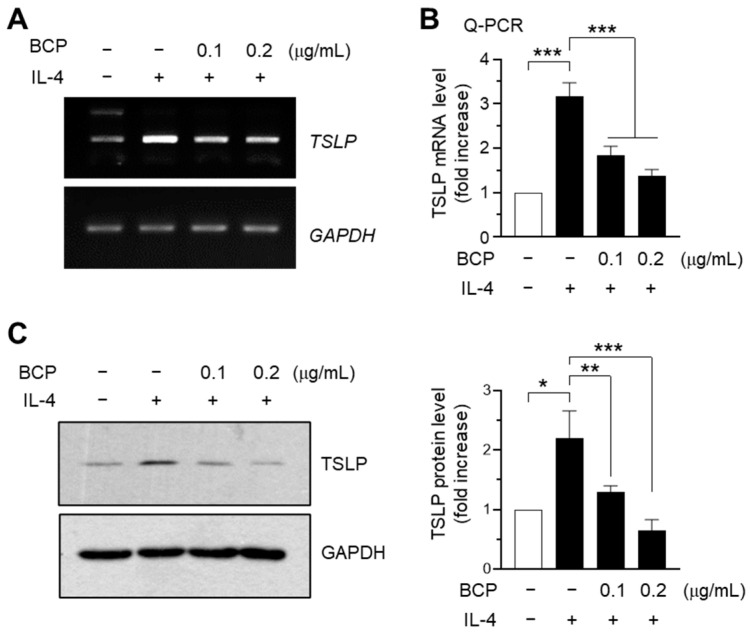
Effect of BCP on the inhibition of TSLP expression in HaCaT keratinocytes. (**A**) HaCaT cells treated with BCP (0.1 and 0.2 μg/mL) for 30 min were incubated with 20 ng/mL IL-4 for 12 h. The TSLP mRNA levels were measured using RT-PCR. GAPDH, internal control. (**B**) TSLP mRNA levels were quantified using Q-PCR. The relative expression level was normalized to the GAPDH level, and data are presented as mean ± SD (*n* = 3). *** *p* < 0.001. (**C**) HaCaT cells were pretreated with BCP (0.1 and 0.2 μg/mL) for 30 min, followed by the addition of 20 ng/mL IL-4 for 24 h. The expression level of TSLP protein was measured by immunoblot analysis. Each band intensity was normalized to the GAPDH level, and the value was expressed as a fold increase over the control group using ImageJ software. Error bar, mean ± SD (*n* = 3). * *p* = 0.001, ** *p* < 0.006, *** *p* < 0.001.

As keratinocyte-derived TSLP plays a crucial role in the initiation and progression of AD, TSLP is considered a druggable target for treating AD [22]. To investigate whether BCP affected TSLP expression, we used an immortalized HaCaT keratinocyte cell line. IL-4 is a cytokine that induces the differentiation of naive Th0 cells to Th2 lymphocytes. RT-PCR analysis showed that IL-4 enhanced TSLP mRNA expression, which was abrogated by BCP in a dose-dependent manner (Figure 3A). The BCP-induced decrease in TSLP mRNA levels was quantified by quantitative real-time PCR (Q-PCR). IL-4 increased TSLP mRNA levels by 3.17 ± 0.306-fold compared to the control group. Notably, the TSLP mRNA levels decreased by 1.83 ± 0.208- and 1.37 ± 0.153-fold, respectively, compared to the levels in the control group upon treatment with 0.1 and 0.2 μg/mL BCP (Figure 3B). BCP consistently suppressed the IL-4-induced TSLP protein accumulation in a dose-dependent manner (Figure 3C). These data suggested that BCP inhibited IL-4-induced TSLP expression at the mRNA level in HaCaT keratinocytes.

### 2.4. Chrysin EGR1 Is Involved in the BCP-Induced Suppression of TSLP Expression Evoked by IL-4

**Figure 4:**
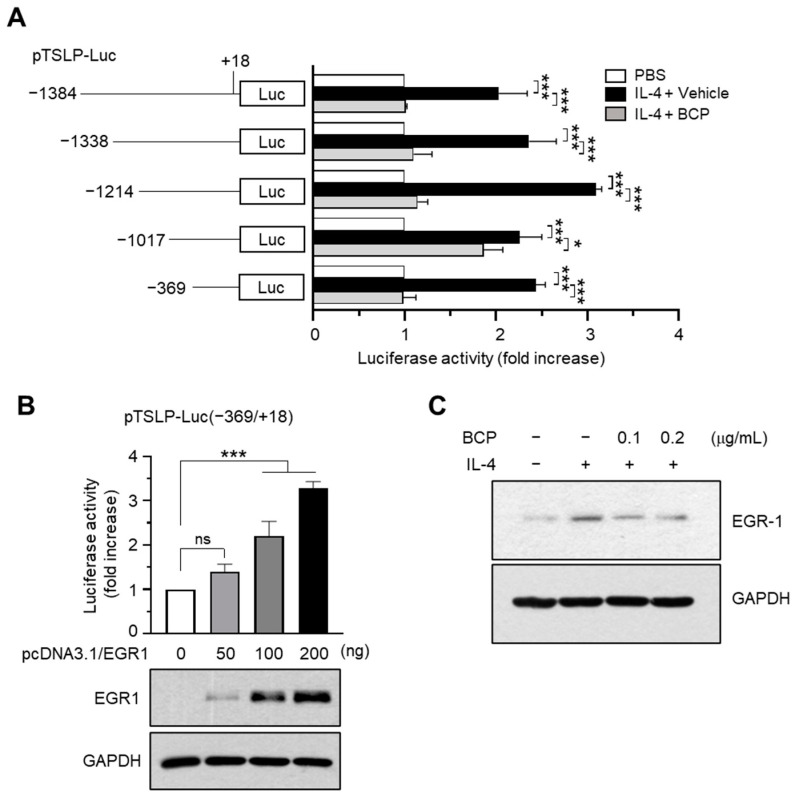
Effect of BCP on the suppression of TSLP promoter activity. (**A**) Luciferase assay using a serial deletion construct of the TSLP promoter-reporters. Data are expressed as the mean ± SD (*n* = 3). * *p* = 0.045; *** *p* < 0.0001. (**B**) TSLP promoter-reporter (pTSLP-Luc(−369/+18) were co-transfected with EGR1 expression plasmids (pcDNA3.1/EGR1). After 48 h, luciferase reporter activity was measured. The expression levels of the transfected EGR1 are shown at the bottom of the graph. GAPDH was used as an internal control. Error bar, mean ± SD (*n* = 3). ns, not significant (*p* = 0.110); *** *p* < 0.001. (**C**) Immunoblot analysis after treatment of HaCaT cells with BCP (0.1 and 0.2 μg/mL) for 30 min and 20 ng/mL IL-4 for 1 h. GAPDH was used as an internal control.

TSLP mRNA expression is regulated by many transcription factors, such as NF-κB and EGR1 [6]. To identify the transcription factor responsible for the suppression of *TSLP* expression by BCP, we used a series of TSLP promoter deletion constructs [23]. IL-4-induced TSLP promoter-reporter activity was efficiently reduced by BCP treatment in all constructs, suggesting that the BCP response element was located between nucleotides −369 and +18 (Figure 4A). 

Previously, we demonstrated that the EGR1-binding sequence (EBS) located at the –206/−187 position is involved in tumor necrosis factor-alpha (TNFα)-induced TSLP promoter activation [23]. EGR1 is a family of inducible transcription factors containing the DNA binding domains of the Cys2-His2-type zinc finger. It is involved in the regulation of immune responses [24]. In the skin, EGR1 mediates TNFα-induced inflammatory cytokines, including TSLP, and Egr1 deficiency ameliorated AD-like skin inflammation in a mouse model [25]. These findings suggest that EGR1 is crucial for cutaneous inflammatory response through TSLP expression in inflammatory skin conditions. We also confirmed that transient transfection with EGR1 expression plasmids (pcDNA3.1/Egr1) increased the luciferase activity of the −369/+18 promoter construct in a concentration-dependent manner (Figure 4B). Moreover, IL-4 stimulated EGR1 protein accumulation, which was abrogated in a BCP concentration-dependent manner (Figure 4C). These data suggested that BCP inhibited the IL-4-induced TSLP expression by downregulating the EGR1 expression.

### 2.5. Role of MAPK Pathways in IL-4-Induced EGR1 Expression

**Figure 5:**
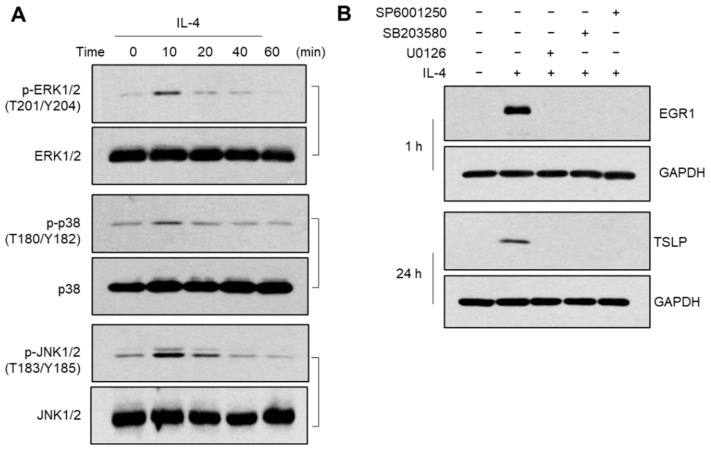
Effect of IL-4 on the activation of MAPK pathways. (**A**) After treatment with IL-4 (20 ng/mL), immunoblotting was performed using whole-cell lysates. (**B**) HaCaT cells were treated with 10 µM U0126, 20 µM SB203580, or 20 µM SP600125 for 30 min and then treated with 20 ng/mL IL-4 for 1 h or 24 h. Whole-cell lysates were immunoblotted with antibodies to EGR1 and TSLP.

Mitogen-activated protein kinases (MAPKs) mediate EGR1 expression in a lot of cell types [26]. In keratinocytes, IL-4 stimulated the phosphorylation of extracellular signal-regulated kinase (ERK), stress-activated protein kinase/c-jun N-terminal kinase (JNK), and p38 kinase within 10 min of IL-4 treatment (Figure 5A), suggesting the activation of all three major MAPKs by IL-4. To determine which MAPKs are involved in EGR1 and TSLP expression, we treated the cells with IL-4 in the presence or absence of MAPK inhibitors (U0126 for ERK inhibition, SB600125 for p38 kinase inhibition, and SP600125 for JNK inhibition) for 1 and 24 h. The treatment with MAPK inhibitors almost blocked the IL-4-induced accumulation of EGR1 and TSLP proteins (Figure 5B), suggesting that all three MAPKs are involved in IL-4-induced EGR1 and TSLP expression. 

### 2.6. BCP Inhibits All Three MAPKs Activated by IL-4

**Figure 6:**
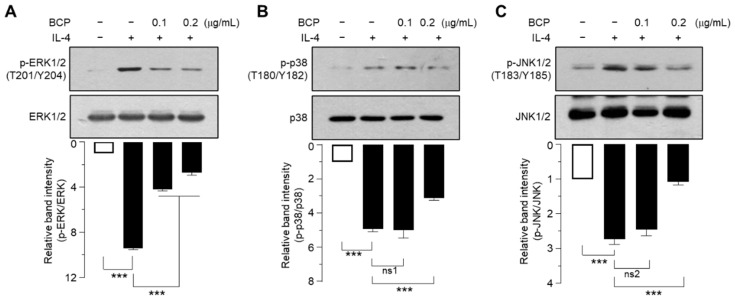
Effect of BCP on the inhibition of IL-4-induced MAPK pathways. HaCaT cells were treated with BCP (0.1 and 0.2 μg/mL) for 30 min and then treated with 20 ng/mL IL-4 for 1 h. Immunoblotting was performed using anti-phospho-ERK1/2 (T201/Y204) (**A**), p-p38 (T180/Y182) (**B**), and p-JNK1/2 (T183/Y185) antibody (**C**). Total ERK2, p38, or JNK2 was used as an internal control. The band intensities of phosphorylated MAPK proteins were normalized to the total proteins corresponding to each MAPK protein using ImageJ software. Error bar, mean ± SD values (*n* = 3). ns1, *p* = 0.981; ns2, *p* = 0.054; *** *p* < 0.001.

We then investigated whether BCP inhibits IL-4-induced MAPK activation. We found that 0.2 μg/mL of BCP significantly (*p* < 0.001 in all cases) inhibited IL-4-induced phosphorylation of ERK1/2 (Figure 6A), p38 kinase (Figure 6B), and JNK1/2 MAPKs (Figure 6C). Notably, BCP inhibited ERK1/2 phosphorylation more effectively than other MAPKs, suggesting that while IL-4 activates the three major MAPKs, BCP differentially inhibits MAPK signaling to downregulate EGR1 expression.

### 2.7. The Topical Application of BCP Inhibits EGR1 and TSLP Expression Induced by IL-4 in AD-like Skin Lesions In Vivo

**Figure 7:**
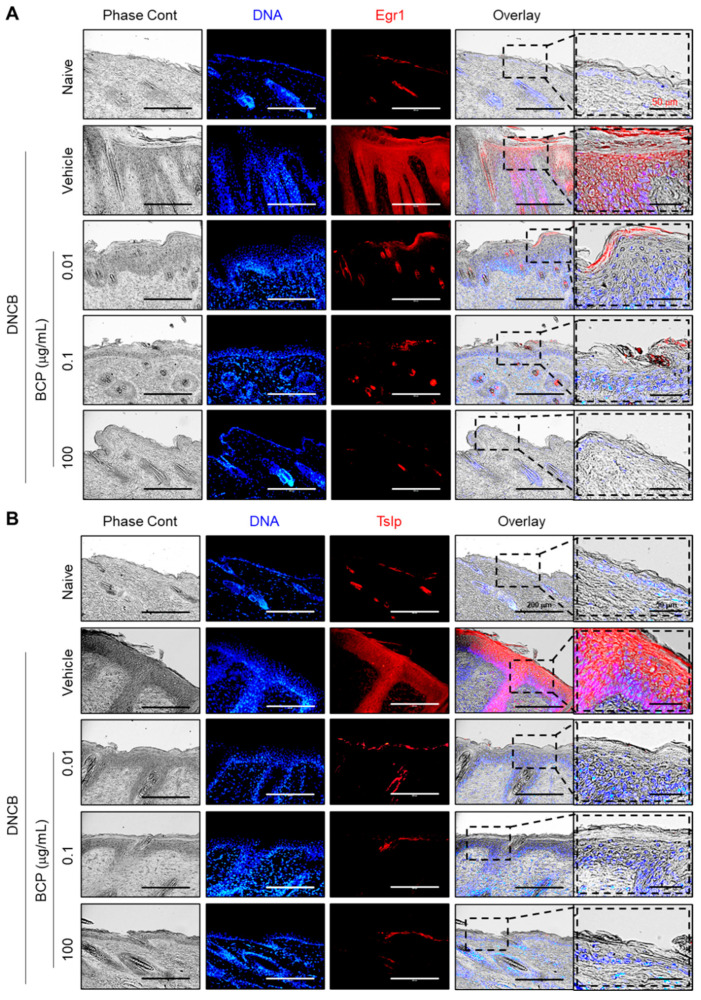
Effect of topical administration of BCP on EGR1 and TSLP expression in DNCB-induced inflamed skin tissues (**A**,**B**) BALB/c mice were either untreated (naive), treated with DNCB + vehicle (PBS), or DNCB + BCP (0.001, 0.1, and 100 μg/mL). Paraffin-embedded tissue sections were immunostained with EGR1 (**A**) and TSLP antibody (**B**). Rhodamine red-X-conjugated antibody was used as a secondary antibody (red fluorescence). Hoechst 33258 was used for counterstaining nuclear DNA (blue fluorescence). Scale bars, 200 μm or 50 μm (enlarged images). The area of the dashed box is enlarged in the right panel.

To verify the effects of BCP on EGR1 and TSLP expression in vivo, we induced AD-like skin lesions by DNCB in BALB/c mice. The presence of EGR1 and TSLP in mouse skin tissues was visualized by immunofluorescence staining. Positive staining for EGR1 (Figure 7A) and TSLP (Figure 7B) remarkably increased throughout the epidermal layer of DNCB-treated mice. Notably, the topical application of BCP dose-dependently reduced the positive staining for both EGR1 and TSLP, reducing the abnormally thick epidermal layer. These data suggest that EGR1 is crucial in TSLP expression in inflamed mouse skin, and aberrant expression of EGR1 and TSLP is associated with the severity of pathological manifestations. 

## 3. Discussion

Various systemic and topical medications have been developed to treat AD [27]. Immunosuppressants, such as corticosteroids, calcineurin inhibitors, and Janus kinase (JAK) inhibitors, improve moderate-to-severe AD. However, potentially serious side effects limit their long-term use. The CB2 receptor exhibits anti-inflammatory properties [16]. BCP is a natural agonist of the CB2 receptor [18]. BCP has been identified in many plants, including *Copaifera langsdorffii* (diesel tree), *Chromolaena odorata* (Siam weed or Christmas bush), *Humulus lupulus* (common hop), *Piper nigrumand* (black pepper), and *Syzygium aromaticum* (clove) [28]. It is also found in *Cymbopogon nardus* (citronella grass), Pinus (pine tree), *Chenopodium ambrosioides* (Mexican-tea), *Cannabis sativa* (hemp), in plants of the genus Copaifera (e.g., Balsam), Artemisia (e.g., Mugworts), Murraya (e.g., orange jessamine), Cordia (e.g., manjack), Spiranthes (e.g., lady’s tresses), Ocimum (e.g., holy basil), Croton (rushfoil), and in the leaves of Annona (e.g., custard apple) [28], and medicinal herbs such as *Ageratum houstonianum* [29].

Several studies have demonstrated that CREB-binding protein inhibits the lipopolysaccharide-induced TNF-α and IL-1β expression in primary monocytes/macrophages [18] and exerts broad anti-inflammatory properties in vivo [19,20]. Furthermore, BCP has an excellent safety profile [30] and is approved for human use by the United States Food and Drug Administration (USFDA) as a food additive (CFR—Code of Federal Regulations Title 21; approval no. 21CFR172.515 available at: https://www.accessdata.fda.gov/scripts/cdrh/cfdocs/cfcfr/CFRSearch.cfm?fr=172.515&SearchTerm=caryophyllene, accessed on 16 November 2022), implying that BCP exerts potent pharmacological properties and therapeutic benefits [31]. However, despite the beneficial effects of BCP in modulating inflammatory responses and its excellent safety profile, the therapeutic potential of BCP in AD remains unexplored. As BCP possesses potent skin penetration properties [32], we evaluated the in vivo efficacy of the topical administration of BCP in treating AD using a clinically relevant mouse model challenged with DNCB. We observed that topical BCP substantially reduced papilliform epidermal hyperplasia and the population of TB-stained cells, suggesting that BCP inhibited the infiltration of inflammatory cells and alleviated AD-like skin inflammation by DNCB. 

The hyperactivation of Th2 responses is a hallmark of AD pathogenesis [2]. TSLP is a master regulator of the Th2 immune response in the skin [8]. To understand the molecular action of BCP underlying the improvement in AD-like skin inflammation, we focused on the effect of BCP on TSLP expression in keratinocytes. BCP inhibited the TSLP expression in IL-4-stimulated HaCaT cells, as revealed by promoter-reporter activity and mRNA and protein expression levels, suggesting that BCP abrogates IL-4-induced TSLP expression at the transcriptional level. Previously, we demonstrated that chrysin (5,7-dihydroxyflavone) [23] and saikosaponins [33] inhibit TNFα-induced *TSLP* promoter activity by inhibiting MAPK-dependent EGR1 expression. Similarly, BCP inhibited the IL-4-induced phosphorylation of ERK1/2, JNK1/2, and p38 kinase and reduced the IL-4-induced *TSLP* expression by inhibiting MAPK-mediated EGR1 expression. Previous studies have demonstrated that BCP inhibits lipopolysaccharide-induced IL-1β, IL-6, and TNFα production in THP-1 human monocytic cells [34] and prevents high glucose-induced oxidative stress and inflammation through the inhibition of NF-κB and Nrf2 in mesangial cells [35]. Moreover, BCP reduces proinflammatory cytokine TNFα, IL-6, and IL-12 production through the inhibition of NLRP3 inflammasome in human peripheral blood mononuclear cells [36], and alleviated lipopolysaccharide-induced lung inflammation through the inhibition of transforming growth factor β-activated kinase 1 (TAK1)/MAPK-mediated signaling pathways [20]. These findings suggest that BCP exhibits anti-inflammatory features in various cells and tissues by targeting NF-κB-mediated pathways. In addition to NF-κB, our findings further support the anti-inflammatory effect of BCP in suppressing TSLP expression by inhibiting MAPK-dependent EGR1 expression in an inflammatory skin environment. 

**Figure 8:**
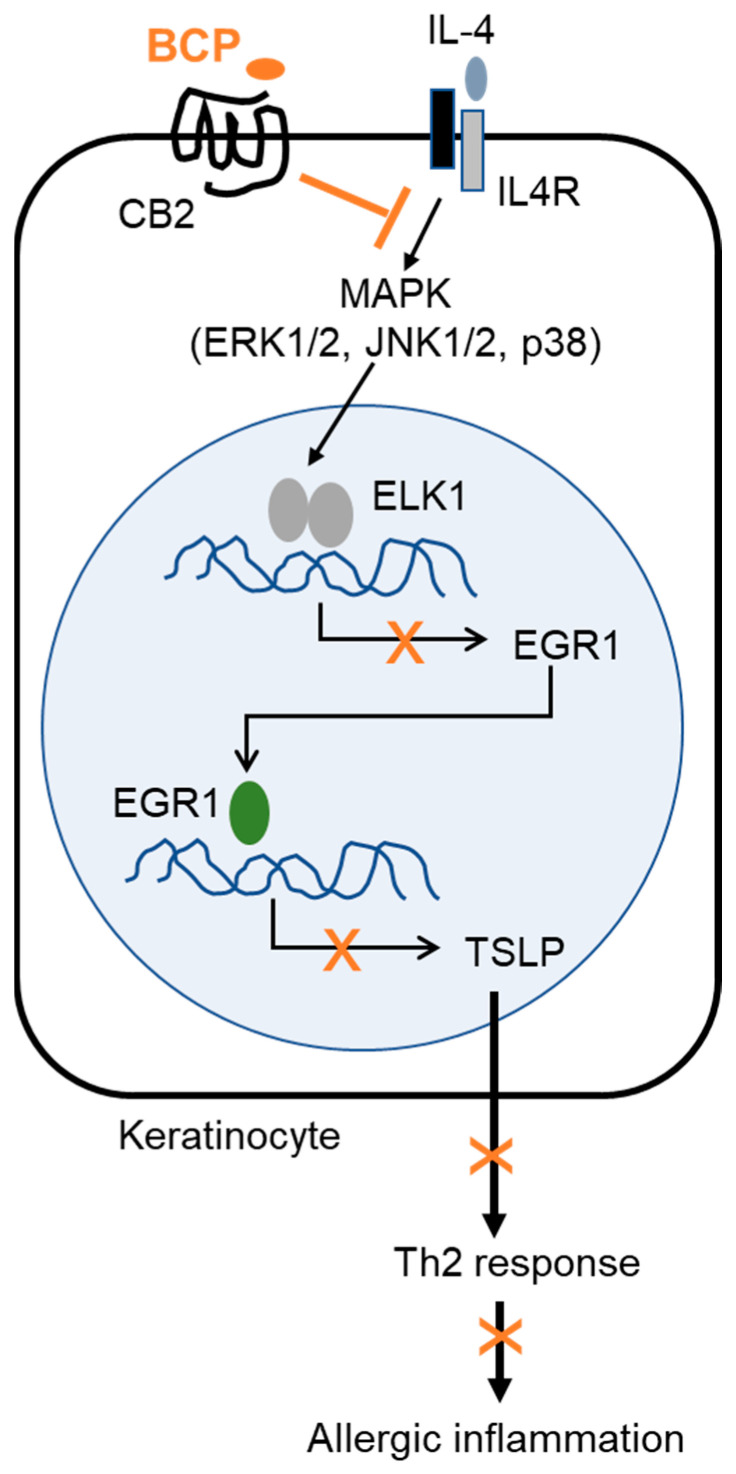
A hypothetical model for the therapeutical efficacy of BCP on allergic inflammatory skin diseases. BCP, β-caryophyllene; CB2, cannabinoid receptor 2; IL-4, interleukin 4; IL4R, IL-4 receptor; DNCB, 2,4-dinitrochlorobenzene; MAPK, mitogen-activated protein kinase; ERK, extracellular signal-regulated kinase; JNK, c-Jun N-terminal kinase; ELK1, ETS domain-containing protein like-1; EGR1, early growth response 1; TSLP, thymic stromal lymphopoietin; Th2, T helper cell 2.

Moreover, BCP enhances the remodeling of rat wound skin in vivo through antioxidant, anti-inflammatory, and re-epithelization activities [37], suggesting that BCP has potential medicinal skin wound-healing and anti-inflammatory properties. In conclusion, the topical application of BCP could relieve the inflammatory response through, at least in part, the downregulation of EGR1-mediated TSLP expression in human AD patients (Figure 8). Therefore, BCP may be applicable for the development of topical therapeutic agents for chronic skin inflammatory diseases, such as AD. 

## 4. Materials and Methods

### 4.1. Materials

Materials used in this study are described in the Supplementary Materials.

### 4.2. Induction of Atopic Dermatitis-like Skin Inflammation on the Dorsal Skin of Mice

AD-like inflammation on the dorsal skin of BALB/c mice was induced by DNCB, as described previously [23]. The detailed experimental procedure is described in the Supplementary Materials. Animal experiments were performed under the guidelines for animal experiments and procedures approved by the Konkuk University Institutional Animal Care and Use Committee (IACUC; approval number KU22011). 

### 4.3. Histological Analysis

Hematoxylin and eosin (H&E) and toluidine blue (TB) staining were performed as previously described [38]. 

### 4.4. Cells

HaCaT cells (Cell Line Service, Eppelheim, Germany) were cultured in Dulbecco’s modified Eagle’s medium supplemented with 10% fetal bovine serum (HyClone, Logan, UT, USA) and penicillin-streptomycin (Sigma-Aldrich, St. Louis, MO, USA).

### 4.5. Reverse Transcription-PCR (RT-PCR) 

RT-PCR was performed as previously described [23]. The PCR amplicons were separated on 2% agarose gel electrophoresis. PCR primer sequences and PCR conditions are described in the Supplementary Materials.

### 4.6. Quantitative Real-Time PCR (Q-PCR)

TSLP mRNA expression levels were quantified, as described previously [23]. Detailed Q-PCR primers and experimental procedures for Q-PCR are described in the Supplementary Materials.

### 4.7. TSLP Promoter-Reporter Assay

Generation of the human TSLP promoter-reporters and luciferase promoter-reporter assays have been described elsewhere [23]. The promoter-reporter activity was expressed relative to the luciferase activity of untreated cells. Luminescence was measured using Centro LB960 (Berthold Tech, Bad Wildbad, Germany).

### 4.8. Immunoblot Analysis

Immunoblotting was performed, as described previously [23]. In some experiments, immunoreactive intensities were quantitated using ImageJ version 1.52a (National Institute of Health, Bethesda, MD, USA) and normalized to GAPDH intensities. 

### 4.9. Immunofluorescence Staining

Immunofluorescence staining of skin sections was performed as previously described [23]. Antibodies used in this study and experimental procedures are described in the Supplementary Materials.

### 4.10. Statistical Analysis

Data are presented as the mean ± standard deviation (SD). Statistical significance was analyzed using GraphPad Prism (version 9.0.1; GraphPad Software, Inc., La Jolla, CA, USA). Statistical significance was set at *p*-value ≤ 0.05.

## 5. Conclusions

Topical BCP ameliorated AD-like skin lesions in BALB/c mice. Mechanistically, BCP downregulates IL-4-stimulated TSLP expression by downregulating MAPK-mediated EGR1 expression in keratinocytes. These findings suggest that BCP has therapeutic potential and feasibility as a source for topical drug development against cutaneous inflammatory diseases, such as AD.

## Data Availability

Not applicable.

## Supplementary Materials

The following are available online at https://www.mdpi.com/article/10.3390/ijms232314861/s1.

## Abbreviations

AD	atopic dermatitis
BCP	β-caryophyllene
CB	cannabinoid receptor
DNCB	2,4-dinitrochlorobenzene
EGR1	early growth response 1
ERK	extracellular signal-regulated kinases
GAPDH	glyceraldehyde 3-phosphate dehydrogenase
H&E	hematoxylin and eosin
IL	interleukin
JNK	c-Jun N-terminal kinase
MAPK	mitogen-activated protein kinases
Q-PCR	quantitative real-time PCR
RT-PCR	reverse transcription polymerase chain reaction
TB	toluidine blue
Th2	T helper cell 2
TNFα	tumor necrosis factor-alpha
TSLP	thymic stromal lymphopoietin
