# Supplementary material for: β-Caryophyllene Ameliorates 2,4-Dinitrochlorobenzene-Induced Atopic Dermatitis through the Downregulation of Mitogen-Activated Protein Kinase/EGR1/TSLP Signaling Axis
Source: Int J Mol Sci. 2022 Nov 28;23(23):14861. doi: 10.3390/ijms232314861 (PMC9740728; doi:10.3390/ijms232314861)
Supplement: Supplementary file 1 [file ijms-23-14861-s001.zip › ijms-2039412-supplementary.pdf]

β-Caryophyllene ameliorates 2,4-dinitrochlorobenzene-induced atopic dermatitis through the downregulation of mitogen-activated protein kinase/EGR1/TSLP signaling axis

Sung Shin Ahn[1,‡], Hyunjin Yeo[1,‡], Euitaek Jung[1], Sukjin Ou[1], Young Han Lee[1], Yoongho Lim[2] and Soon Young Shin[1,*]

[1] Department of Biological Sciences, Sanghuh College of Lifesciences, Konkuk University, Seoul 05029, Republic of Korea
[2] Division of Bioscience and Biotechnology, Konkuk University, Seoul 05029, Republic of Korea
* Correspondence: Correspondence: shinsy@konkuk.ac.kr; Tel.: +82-2-2030-7946
‡ S.S.A. and H.Y. contributed equally.

Supplementary Materials

Materials

β-caryophyllene, 2,4-dinitrochlorobenzene, H&E staining kit, and toluidine blue were purchased from Sigma-Aldrich (St. Louis, MO, USA). IL-4 was purchased from ProSpec (ProSpec-Tany TechnoGene). The firefly luciferase assay system was obtained from Promega (Madison, WI, USA). The anti-TSLP antibody was obtained from Novus Biologicals (Centennial, CO, USA), and phospho-ERK1/2 (Thr202/Tyr204), phospho-p38 (Thr180/Tyr182), and phospho-JNK1/2 (Thr183/Tyr185) antibodies were obtained from Cell Signaling Technology (Danvers, MA, USA). Anti-GAPDH and anti-EGR-1 antibodies were purchased from Santa Cruz Biotechnology (Dallas, TX). Immunofluorescent secondary antibodies conjugated to rhodamine red-X were obtained from Jackson ImmunoResearch Laboratories (West Grove, PA, USA). Male BALB/c mice (7–8 weeks old, 20–25 g body weight) were purchased from Orient Bio Inc. (Seongnam, Korea).

Induction of atopic dermatitis-like skin inflammation on the dorsal skin of mice

Male BALB/c mice (7–8 weeks old, 20–25 g body weight) were purchased from Orient Bio Inc. (Seongnam, Korea). Mice were housed under standard laboratory conditions in a pathogen-free environment. The mice were randomly divided into five groups (n = 5 in each group): Group I, naive; Group II, DNCB + vehicle (acetone/olive oil (1:3, v/v) + 0.1% dimethyl sulfoxide); Group III, DNCB + 0.01 μg/mL BCP; Group IV, DNCB + 0.1 μg/mL BCP; Group V, DNCB + 100 μg/mL BCP. DNCB-induced AD-like inflammation in the dorsal skin was induced as described previously [23]. Briefly, all mice, except for the naive group, were sensitized with 4% SDS on the dorsal skin and challenged with 1% DNCB dissolved in an acetone:olive oil mixture (1:3, v/v). BCP powder was dissolved in dimethyl sulfoxide to prepare a stock solution (100 mg/mL) and then diluted using acetone:olive oil mixture (1:3, v/v) to final concentrations of 0.01, 0.1, and 100 μg/mL. On day 20, all mice were sacrificed, and paraffin-embedded tissue sections were prepared.

Primers for RT-PCR

Total RNA was isolated using a TRIzol RNA Extraction Kit (Invitrogen, Carlsbad, CA, USA), and cDNA was prepared using an iScript cDNA Synthesis Kit (Bio-Rad, Hercules, CA, USA). The sequence-specific PCR primers used were as follows:

- TSLP forward, 5-′-TAG CAA TCG GCC ACA TTG CCT-3′
- TSLP reverse, 5′-GAA GCG ACG CCA CAA TCC TTG-3
- GAPDH forward, 5′-CCA AGG AGT AAG AAA CCC TGG AC-3′

- GAPDH reverse, 5′-GGG CCG AGT TGG GAT AGG G-3′

After denaturation at 94 °C for 5 min, the thermal cycling conditions were 30 cycles at 94 °C for 30 s, 58 °C for 30 s, and 72 °C for 1 min. The amplified PCR products were separated using 2% agarose gel electrophoresis and visualized with ethidium bromide.

Primers for quantitative real-time PCR (Q-PCR)

Validated Q-PCR primers and SYBR Green-based fluorescent probes specific for TSLP (id: qHsaCIP0030468) and GAPDH (id: qHsaCEP0041396) were obtained from Bio-Rad. PCR was performed as follows: denaturation at 95 °C for 2 min, followed by 40 cycles 95 °C for 10 s and 60 °C for 45 s. Relative TSLP mRNA levels were normalized to GAPDH using software provided by the manufacturer (Bio-Rad).

Immunofluorescence staining

After deparaffinization and hydration, the tissue sections were incubated overnight with an anti-TSLP (1:100 dilution) or anti-Egr1 (1:100 dilution) antibodies. Immunoreactivity was detected using a fluorescent secondary antibody conjugated with Rhodamine red-X (1:300). Nuclear DNA was counterstained with Hoechst 33258 solution for 10 min. After extensive washing with PBS, the slides were mounted using the ProLong Gold Antifade reagent (Invitrogen). Fluorescent images were captured using an EVOS FL fluorescence microscope (Advanced Microscopy Group, Bothell, WA, USA).
